# Autoantibodies against AT1 Receptor Contribute to Vascular Aging and Endothelial Cell Senescence

**DOI:** 10.14336/AD.2018.0919

**Published:** 2019-10-01

**Authors:** Meili Wang, Xiaochen Yin, Suli Zhang, Chenfeng Mao, Ning Cao, Xiaochun Yang, Jingwei Bian, Weiwei Hao, Qian Fan, Huirong Liu

**Affiliations:** ^1^Department of Physiology and Pathophysiology, School of Basic Medical Sciences, Capital Medical University, Beijing, China.; ^2^Beijing Key Laboratory of Metabolic Disorders Related Cardiovascular Disease, Capital Medical University, Beijing, China.; ^3^Department of Physiology and Pathophysiology, School of Basic Medical Sciences, Peking University Health Science Center, Beijing, China.; ^4^Key Laboratory of Molecular Cardiovascular Science, Ministry of Education, Beijing, China.; ^5^Beijing Anzhen Hospital, Capital Medical University, Beijing, China.

**Keywords:** AT1 receptor, Autoantibody, Peripheral arterial disease, Vascular aging, EC senescence

## Abstract

Vascular aging predisposes the elderly to the progression of many aging-related vascular disorders and leads to deterioration of cardiovascular diseases (CVD). However, the underlying mechanisms have not been clearly elucidated. Agonistic autoantibodies against angiotensin II type 1 (AT1) receptor (AT1-AAs) have been demonstrated to be pro-inflammatory and contribute to the progression of atherosclerosis. However, the association between AT1-AAs and vascular aging has not been defined. Peripheral arterial disease (PAD) is an acknowledged vascular aging-related disease. In this study, AT1-AAs were detected in the sera of patients with PAD and the positive rate was 44.44% (n=63) *vs.* 17.46% in non-PAD volunteers (n=63). In addition, case-control analysis showed that AT1-AAs level was positively correlated with PAD. To reveal the causal relationship between AT1-AAs and vascular aging, an AT1-AAs-positive rat model was established by active immunization. The carotid pulse wave velocity was higher, and the aortic endothelium-dependent vasodilatation was attenuated significantly in the immunized rats. Morphological staining showed thickening of the aortic wall. Histological examination showed that levels of the senescent markers were increased in the aortic tissue, mostly located at the endothelium. In addition, purified AT1-AAs-IgGs from both the immunized rats and PAD patients induced premature senescence in cultured human umbilical vein endothelial cells. These effects were significantly blocked by the AT1 receptor blocker. Taken together, our study demonstrates that AT1-AAs contribute to the progression of vascular aging and induce EC senescence through AT1 receptor. AT1-AA is a novel biomarker of vascular aging and aging-related CVD that acts to accelerate EC senescence.

Vascular aging is a dominant risk factor for many aging-related cardiovascular diseases (CVD), represented by atherosclerosis, coronary artery disease, vascular calcification, hypertension and stroke [1]. Many structural and functional changes are involved in this process, including endothelial dysfunction, vascular stiffening, increased secretion of inflammatory factors, and accumulation of oxidative stress [2]. Especially, vascular aging is characterized by endothelial dysfunction, which is associated with decreased endothelium-dependent relaxation during aging in humans [3]. Stiffening of large elastic arteries is recognized as another key vascular change associated with the development of endothelial dysfunction during aging [4, 5]. Senescent vascular cells, particularly senescent endothelial cells (ECs), impair the vascular function and contribute to aging-related vascular diseases [6, 7]. But how genetic and environmental factors influence ECs and induce senescence remains largely unknown.

As a key regulator of vascular physiology, the renin-angiotensin system (RAS) has been implicated in the development and progression of vascular aging [8]. Interruption of the RAS pathway, either by preventing the formation of angiotensin II (Ang II) or by blocking the Ang II type 1 (AT1) receptor, has been proven to be highly successful in retarding vascular aging phenotypes [9, 10]. Meanwhile, inappropriate activation of the RAS, independent of the classic bioactive molecule Ang II, may cause excessive activation of the AT1 receptor and induce chronic inflammation [11, 12], but how this occurs is not fully understood.

At the end of the twentieth century, a specific autoantibody against AT1 receptor (AT1-AA) has been discovered and was found to exist in patients with preeclampsia [13], malignant hypertension [14], refractory hypertension [15] and renal-allograft rejection [16]. AT1-AAs could specifically bind to the second extracellular loop of AT1 receptor and were found to have a receptor agonist-like effect [13]. AT1-AAs were proven to be pro-inflammatory via the transcription factor nuclear factor-kappa B (NF-κB) pathway, thus enhancing the expression of inflammatory factors in ECs [17]. Moreover, we have previously demonstrated that AT1-AAs induced endothelial damage and contributed to endothelial dysfunction *in vivo* [18]. Most importantly, AT1-AAs have been reported to accelerate aortic atherosclerosis in mice [19]. In a recent study, Peter M and colleagues demonstrated that higher AT1-AAs level was associated with inflammation, hypertension and adverse outcomes [20]. All the above evidence suggests a close relationship between AT1-AAs and vascular aging. Nevertheless, whether AT1-AAs can induce vascular aging or EC senescence has never been explored.

To address this issue, we performed a case-control study to explore the association between serum AT1-AAs levels and peripheral arterial disease (PAD). Next, we conducted *in vivo* experiments with the use of AT1-AAs-positive rat models to explore the role of AT1-AAs in inducing vascular aging. Further, we performed *in vitro* experiments to investigate the effect of AT1-AAs on EC senescence and the underlying mechanism.

## MATERIALS AND METHODS

### Materials

Antibodies against p53, p21, p16^INK4a^ and β-actin were purchased from Abcam (Cambridge, UK). Antibodies against p-eNOS and eNOS were purchased from Cell Signaling Technology (Boston, MA, USA).

### Study population

This study included a total of 126 participants, who were divided into 2 groups: One group was composed of 63 patients with PAD and the other was composed of 63 non-PAD volunteers. The serum samples of both groups were from Beijing Anzhen hospital. PAD was detected by the measurement of ankle-brachial index (ABI) and diagnosed by the presence of an ABI < 0.9. Non-PAD volunteers with a negative medical history of PAD (1.0 < ABI < 1.3) and no evidence of sonographically detectable atherosclerotic diseases served as controls.

This study was approved by the local research ethics committee (Beijing Anzhen hospital, Capital Medical University, Beijing, China). Written informed consents were obtained from all the participants before the study commenced.

### Animals

Healthy male Sprague-Dawley (SD) rats aged 8 weeks (140-160g) were obtained from the Animal Center of Capital Medical University to establish the active immunization model. All the animal experiments followed the guidelines of the Institutional Animal Care and Use Committee and Ethics Committee of Capital Medical University.

### Active immunization

Male SD rats were randomly divided into two groups: the immunized group and the Freund's adjuvant-treated vehicle group. The synthetic peptides corresponding to the sequence of the second extracellular loop of human AT1 receptor (AT_1_R-EC_II_, residues 165-191, sequence I-H-R-N-V-F-F-I-E-N-T-N-I-T-V-C-A-F-H-Y-E-S-Q-N-S-T-L, 95% purity, GL Biochem Ltd, Shanghai) were dissolved in Na_2_CO_3_ solution (100 mM, PH 11.0) to a final concentration of 4 mg/ml and then diluted in normal saline. The antigen solution, together with Freund’s complete adjuvant by an equal proportion, was emulsified and then multiply-injected into the back of the rats subcutaneously (0.4 mg/kg). Booster immunizations were repeated once every two weeks by a single subcutaneous injection, and the antigen was emulsified in Freund’s incomplete adjuvant. The antigen solution was replaced with Na_2_CO_3_ solution following the same procedure in the vehicle group. The blood samples extracted from the tail top of the rats were collected for detection of serum AT1-AAs.

### Enzyme-linked immunosorbent assay (ELISA)

Modified ELISA was used to detect the titers of AT1-AAs in sera as previously described [18]. The results were expressed as OD values measured at 405 nm using a Spectra Max Plus microplate reader (Molecular Devices Corp, Sunnyvale, USA). The positive/negative (P/N) ratio was calculated as follows: (OD of sample - OD of blank control)/(OD of negative control - OD of blank control). Samples with a P/N value >2.1 was considered positive.

### Measurement of pulsed wave velocity (PWV)

The rats were anesthetized with isoflurane and maintained by mask ventilation (3% for induction and 1.5% for maintenance) with a coupled charcoal scavenging system during the measurement. PWV was measured at the distal and proximal locations of the left common carotid artery using a 15-MHz Doppler probe while simultaneously recording the electrocardiogram (ECG) signal. PWV was calculated as a quotient of the separation distance and the transit time between pulse arrivals which was measured from the ECG R-peaks.

### Aortic ring vasodilatory response

As the immunization process finished, rats were anesthetized intraperitoneally with pentobarbital sodium (40 mg/kg). The aorta was isolated from the rats of both vehicle and immunized groups. The dissected vessels were immediately removed and placed in ice-cold Krebs-Henseleit buffer, and then cleaned of additional connective tissues carefully. The aorta was cut into 3-4 mm rings and subsequently subjected to vascular tension experiments. The changes in isometric force were recorded using a PowerLab system (AD Instruments, Australia). The rings were preconstricted with phenylephrine (1 μM). After the contraction reached a plateau, cumulative doses of acetylcholine (ACh, 10^-9^-10^-6^ M) were added to characterize vasorelaxation. To investigate the role of endothelial nitric oxide synthase (eNOS), N-nitro-L-arginine-methyl ester (L-NAME, 100 μM) was used for pretreatment, followed by the continuous addition of sodium nitroprusside (SNP, 10^-9^-10^-6^ M). Vasorelaxation was presented as the percent relaxation, as calculated by the percent decrease in the tension from the phenylephrine-induced preconstriction.

### Preparation of the immunoglobulin G

The blood samples were collected from the abdominal aorta of rats after completion of the immunization process. Total IgGs in the sera of both the rats and human were purified by IgG affinity column (Mab Trap Kit, Amersham) as previously described [18].

### Cell culture and treatment

Human umbilical vein endothelial cells (HUVECs) were purchased from Shanghai Cell Bank, Chinese Academy of Sciences as pooled primary cell lines and cultured with endothelial growth medium (M199, Gibco, San Diego, CA) supplemented with 10% fetal bovine serum (FBS, HyClone, Logan, UT) at 37? in a 5% CO_2_ atmosphere. HUVECs were plated and cultured to 80~90% confluence and treated with various concentrations (0, 0.001, 0.01, 0.1, 1 and 10 μM) of AT1-AAs for 5 days, or a concentration of 1 μM of AT1-AAs, nIgGs, or Ang II for 0, 24, 48 and 72 hrs. In the AT1 receptor blockage study, similarly prepared HUVECs were incubated with valsartan (10 μM) for 2 hrs prior to stimulation with AT1-AAs for 72 hrs.

### Cell cycle assay

The HUVECs were digested with trypsin, collected by centrifugation and fixed with 70% ethanol at 4 ? overnight. Then cells were re-suspended in PBS containing 50 mM propidium iodide and 10 μg/ml RNase for 30 mins at 37 ?. After washing with cold PBS, cell cycles were analyzed by flow cytometric analysis (BD FACS Calibur, Becton Dickinson, East Rutherford, NJ, USA). The number of cells within the G0/G1, S and G2/M phases of the cell cycle was analyzed using ModFit LT, version 2.0 (Verify Software House, Topsham, ME).

### Senescence-associated β-galactosidase (SA β-Gal) assay

The SA β-Gal staining was performed according to the manufacturer’s instructions (Beyotime, China). Briefly, the HUVECs were washed with PBS and fixed with the fixative solution for 15 mins, and then washed and incubated with the staining solution at 37 ? for 16 hrs. Cells were counterstained with DAPI to count the total number and the senescent cells were blue-stained as observed under the light microscope (Olympus, Japan). The SA β-Gal positive cells were counted manually, and the total cell number was quantified using NIH ImageJ software (National Institutes of Health, USA). More than 30,000 cells total were counted in 9 randomly selected fields to determine the percentage of SA β-Gal-positive cells.

### Immunohistochemistry

Tissue sections were prepared and incubated with primary antibodies against p53 (at a dilution of 1:500), p21 (at a dilution of 1:1000) and p16^INK4a^ (at a dilution of 1:1000) overnight at 4 ?. HRP-labeled anti-rabbit or anti-mouse antibodies were applied for 1 hr at room temperature and cell nuclei were visualized with DAPI staining.

### Western blotting

Protein concentrations were evaluated using a BCA Protein Assay Kit (Thermo Scientific, Waltham, MA). Whole cell lysates containing equal amounts of total protein (25 μg) were separated by 8%~15% SDS-PAGE gels and transferred onto PVDF membranes (Millipore, Billerica, MA). The membranes were blocked with 5% milk (in TBST) for 1 hr, and then incubated with corresponding primary antibodies at 4 ? overnight. After washing, the membranes were incubated with secondary antibodies at a 1:20000 dilution and then developed using an ECL regent (Applygen Technologies Inc.).

### RT-qPCR

Total RNA was extracted from cultured HUVECs using the Trizol reagent (Invitrogen, USA), and an equal amount (2 μg) was reverse transcribed into cDNA with the Reverse Transcription System. Quantitation of all gene transcripts was done by qPCR using Power SYBR Green PCR Master Mix (Thermo Scientific, Waltham, MA) and ABI PRISM 7500 sequence detection system (Applied Biosystems, Foster City, CA) with the expression of GAPDH as the internal control. Relative mRNA levels were presented as mean fold change of samples as compared with the control. The primers used are listed in Supplementary Table.

### Statistical methods

All the animal experiments and cell assay data were expressed as mean ± SEM. Differences between two groups were analyzed using Student’s *t*-test. One-way ANOVA followed by Student-Newman-Keuls test was used in pairwise comparison of three or more groups. Chi-square test was used in comparison of rate between two groups. GraphPad Prism 6.0 (GraphPad Software, Inc., San Diego, CA, USA) was used for drafting and statistical analysis; *p* values<0.05 were considered statistically significant.

**Table 1:** Chi-square analysis on demographic characteristics of participants.

Characteristic		PAD		χ^2^	P
		Yes (%)	No (%)		
Gender^△^	male	46 (73.02)	46 (73.02)	—	—
	female	17 (26.98)	17 (26.98)		
Smoking history	Ever	16 (25.40)	10 (15.87)	16.391	<0.001
	Current	34 (53.97)	18 (28.57)		
	Never	13 (20.63)	35 (55.56)		
Drinking history	Ever	9 (14.29)	10 (15.87)	0.184	0.912
	Current	18 (28.57)	16 (25.40)		
	Never	36 (57.14)	37 (58.73)		
Hypertension history	No	15 (23.81)	32 (50.79)	11.097	0.004
	Yes, good drug control	25 (39.68)	12 (19.05)		
	Yes, bad drug control	23 (36.51)	19 (30.16)		
Diabetes	No	42 (66.67)	60 (95.24)	16.676	<0.001
	Yes	21 (33.33)	3 (4.76)		

## RESULTS

### Detection of AT1-AAs in patients with PAD

**Figure 1.**
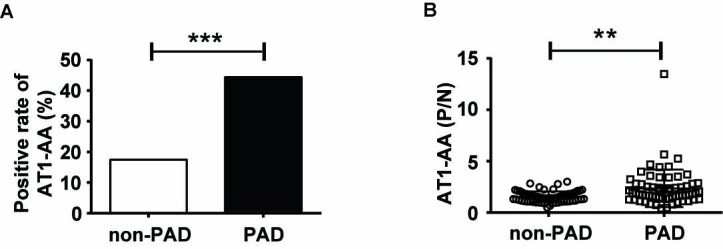
High levels of AT1-AAs in the patients with PAD. A) The positive rate of AT1-AAs in PAD group and the non-PAD group was 44.44% and 17.46%, respectively. (n=63 per group). B) The P/N values of AT1-AAs in the sera samples of PAD patients and non-PAD participants measured by ELISA. PAD, peripheral arterial disease, **p<0.01, ***p<0.001.

Epidemiological studies have shown that PAD, mainly characterized by the presence of increased arterial stiffness, is prevalent in the elderly. Clinically, PAD confers an independent risk for cardiovascular events and total mortality [21, 22]. To assess the association of AT1-AAs with vascular aging-related diseases, we collected sera from 63 PAD patients recruited from Beijing Anzhen hospital and determined the level of AT1-AAs. As shown in Fig. 1A, AT1-AAs were identified as positive in 28 patients, which was significantly higher compared to the non-PAD volunteers as control group (PAD: 44.44% vs CTL: 17.46%, n=63 per group, p<0.001). In addition, the levels of AT1-AAs in PAD patients were generally higher than these in non-PAD volunteers (Fig. 1B).

### Univariate analysis on participants’ characteristics

A hospital based 1:1 matched case-control study was carried out,involving a total of 126 participants. A pair was composed of one PAD patient and one gender- and age-matched non-PAD volunteer. As shown in Table 1, cases (PAD patients) and controls (non-PAD volunteers) did not differ significantly with respect to the proportion whether drinking or not. However, the cases were significantly more likely to have a smoking history, as well as previous clinically diagnosed hypertension and diabetes history.

### Univariate analysis on the biochemical indexes of the participants

**Figure 2.**
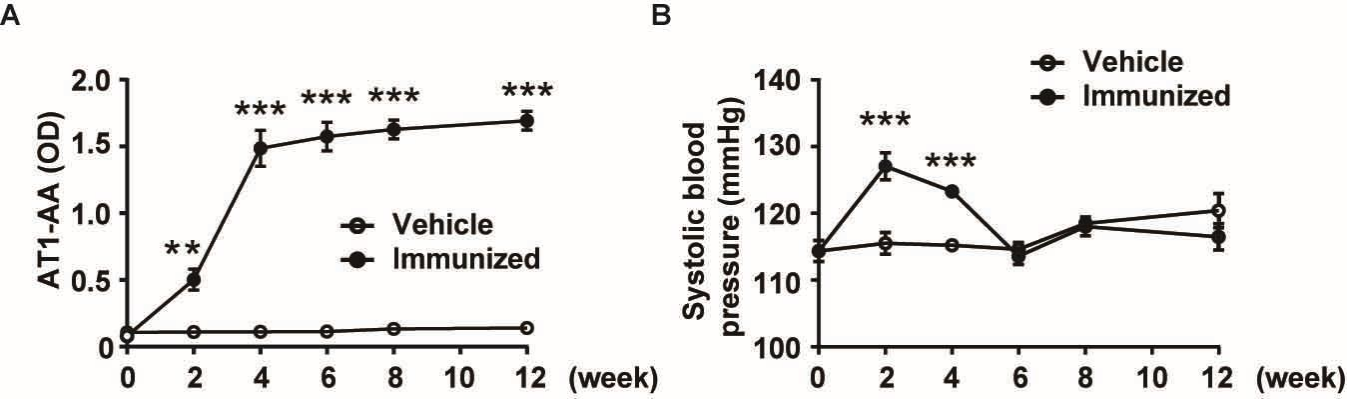
Successful establishment of the AT1-AAs-positive rat model. A) The level of AT1-AAs in sera was detected by ELISA. It increased gradually after the first immunization and reached a peak six weeks later and maintained a high value compared with the vehicle group. B) Blood pressure curves of rats immunized with AT_1_R-EC_II_ peptides or the vehicle group. Data were expressed as means ± SEM, n=8-10 rats per group. ***p<0.001 *vs.* the vehicle group.

### Multivariate analysis of the entire study population

As shown in Table 3, the serum level of AT1-AAs was significantly associated with PAD after adjustment for age, gender, smoking history, diabetes history, hypertension history, TC, TG and LDL levels. The risk for PAD in participants with high levels of AT1-AAs was significantly higher than that in those with low serum levels of AT1-AAs. Specifically, every one unit increase in the P/N value increased the risk of PAD by 3.559-fold (OR=3.559, 95% C.I: 1.638, 7.730).[Table T2-ad-10-5-1012]

**Table 2 T2-ad-10-5-1012:** Wilcoxon rank sum test on the biochemical indexes of PAD patients.

Variables	PAD(n=63)	Non-PAD(n=63)	*Z* value	*P* value
Age^△^	65.29±13.12	64.81±12.94	—	—
AT1-AA(P/N)	2.362± 1.828	1.533± 0.5299	-4.073	<0.001
BMI	23.72± 3.48	24.69± 3.784	-1.506	0.132
Heart rate	80.03± 10.72	77.46± 13.04	-1.347	0.178
SBP	146.3±19.32	142.7±17.35	-1.412	0.158
DBP	78.78±9.868	82.84±13.89	-1.489	0.136
Blood sugar level	5.529± 2.396	5.539± 1.232	-1.629	0.103
TC (mmol/L)	4.608± 1.168	4.171± 1.070	-2.119	0.034
TG (mmol/L)	1.523± 0.8398	1.477± 1.975	-2.499	0.012
HDL (mmol/L)	1.077±0.2667	1.083±0.2648	-0.246	0.805
LDL (mmol/L)	2.956±0.9662	2.582±0.7198	-2.341	0.019

^△^Matching factors;PAD, peripheral arterial disease; BMI, Body Mass Index; TC, Total cholesterol;TG, Triglyceride;HDL, high density lipoprotein;LDL, low density lipoprotein

### Successful establishment of AT1-AAs-positive rat models by active immunization

After demonstrating the close association between the serum level of AT1-AAs and vascular aging-related disease, we sought to determine whether AT1-AAs could induce vascular aging. An AT1-AAs-positive rat model was established by active immunization with the synthetic peptides corresponding to the sequence of the second extracellular loop of human AT1 receptor (AT_1_R-EC_II_). Since the initial immunization, the titers of AT1-AAs in the sera of rats were detected by using the enzyme-linked immunosorbent assay (ELISA) method. As illustrated in Fig. 2A, the serum level of AT1-AAs began increasing from the 2nd week and the concentration of AT1-AAs maintained high until the end of immunization, indicating the successful establishment of the active immunization model. Meanwhile, AT1-AAs were not detected in the vehicle group throughout the entire process. Additionally, we measured the SBP. It was found that SBP began increasing from the 2nd week, remained high until the 4th week, but showed no difference from the 6th week to the end of the experiment (Fig. 2B).

**Figure 3.**
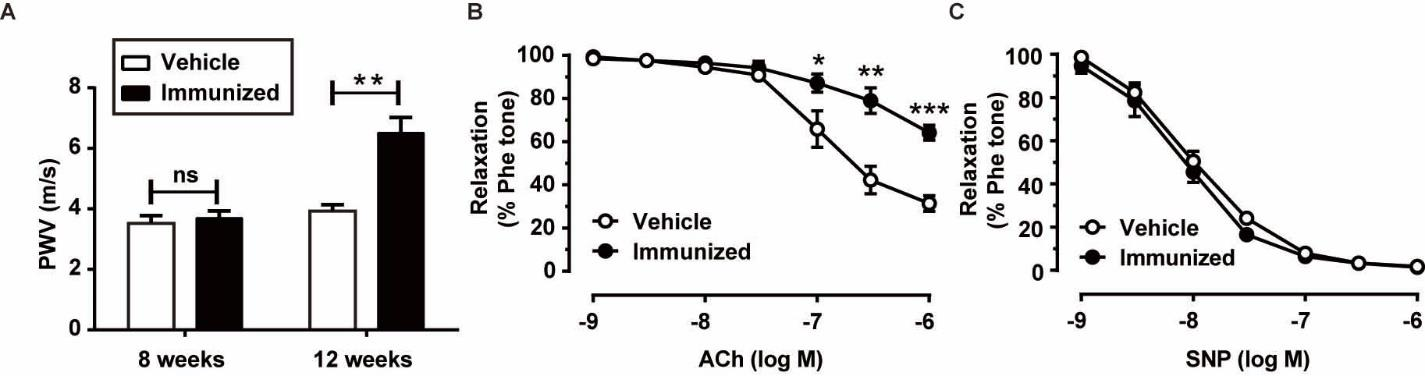
Functional evidence of vascular aging in immunized rats. A) Pulse wave velocity measured in the left common carotid artery from the vehicle and AT_1_R-EC_II_-immunized rats. B) Relaxation curves in response to the vasodilator acetylcholine of phenylephrine (1 μM)-pretreated isolated aortic rings from the vehicle and AT_1_R-EC_II_-immunized rats. C) Relaxation in response to SNP. Isolated aortic rings were treated with L-NAME (100 μM) for 30 mins followed by phenylephrine (1 μM) treatment. Data were expressed as mean ± SEM. n = 6-8 rats for each group, *p < 0.05, **p < 0.01, ***p<0.001 *vs.* the vehicle group.

### AT1-AAs caused vascular aging and endothelial senescence in actively immunized rats

Vascular stiffening is the hallmark of vascular aging, and PWV is considered the gold standard measure of vascular stiffness in humans [23]. In this study, PWV of the rats was measured noninvasively in the left common carotid artery (Supplementary Fig. 1). To determine the earliest timepoint of the occurance of changes in the vascular physiological parameters, we measured the PWV at the 8^th^ and 12^th^ weeks after initial immunization. As shown in Fig. 3A, there was no significant difference in PWV between the vehicle and AT_1_R-EC_II_-immunized groups at the 8^th^ week. However, PWV was increased at the 12^th^ week. To further investigate the association between AT1-AAs and vascular aging, an isometric tension experiment was conducted to evaluate the vasodilatation of the aortas. The aortic rings obtained from the two groups were pre-contracted with 1 μM phenylephrine and then treated with acetylcholine (ACh) (10^-9^-10^-6^ M). As summarized in Fig. 3B, compared with the vehicle group, reduced ACh-induced vasodilatation was observed in the immunized group. Meanwhile, the phosphorylation of endothelial nitric oxide synthase (eNOS) was reduced in the aortic tissues of the immunized rats (Supplementary Fig. 2). These results suggested that AT1-AAs induced dysfunction of endothelium-dependent vasodilatation. Nevertheless, there was no significant difference in sodium nitroprusside (SNP) (10^-9^-10^-6^ M)-induced endothelium-independent vasodilatation between the two groups (Fig. 3C). These data demonstrated that AT1-AAs led to vascular aging-related dysfunction in *vivo*.

**Figure 4.**
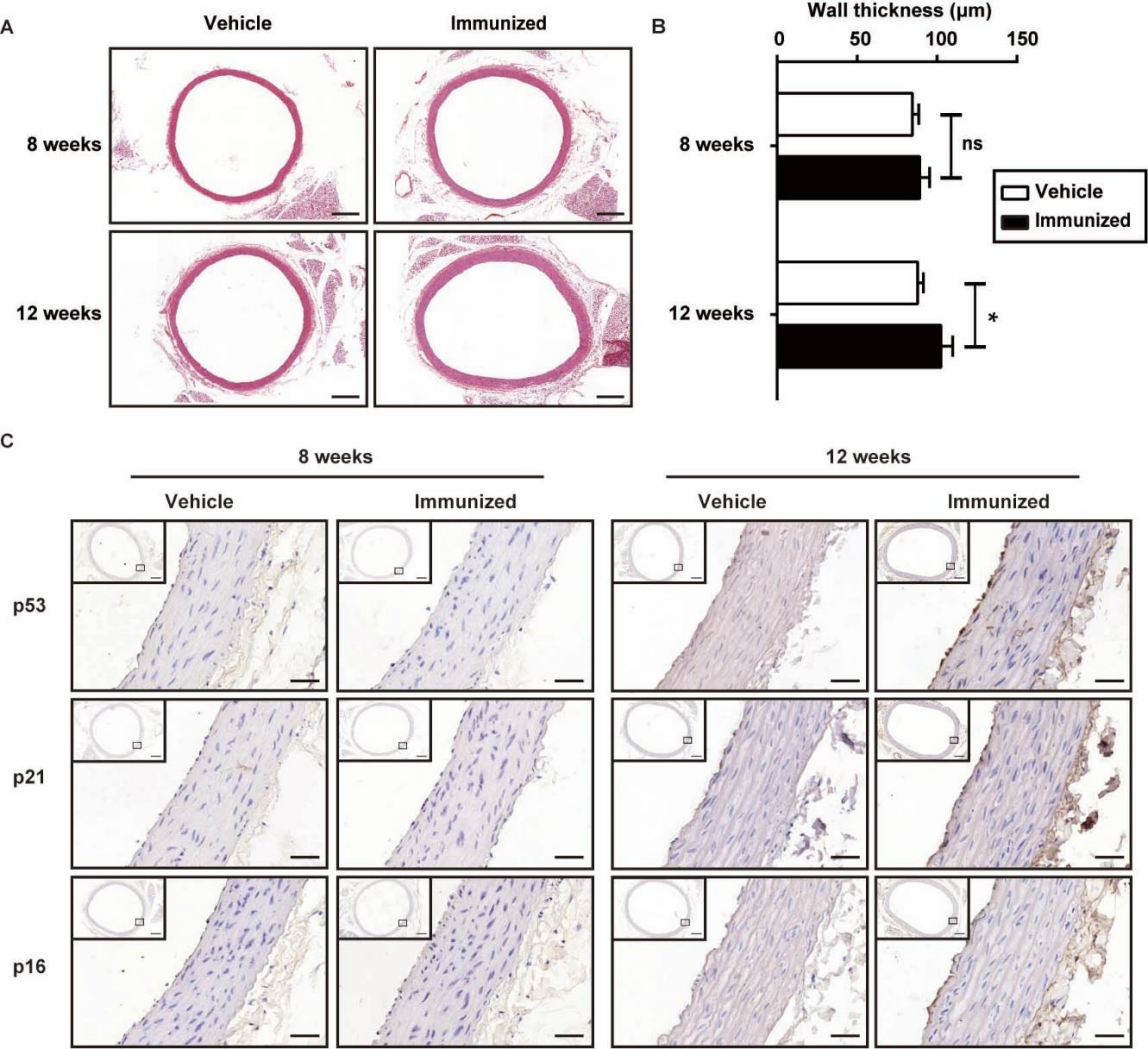
Changes in arterial morphology after immunization. A) Microscopic images of the HE-stained aortic sections. Scale bar = 400 μm. There was no significant difference in arterial wall thickness at the 8^th^ weeks, but it increased significantly after 12-week immunization. B) Quantitative analysis of wall thickness of thoracic aortas at 8 and 12 weeks after immunization. C) Representative images of immunohistochemical staining showing p53, p21 and p16^INK4a^ in the rat aorta of the indicated groups. Scale bar = 400 (small views) and 40 μm (enlarged views). Data were expressed as mean ± SEM. n = 6-8 rats for each group, *p < 0.05.

**Figure 5.**
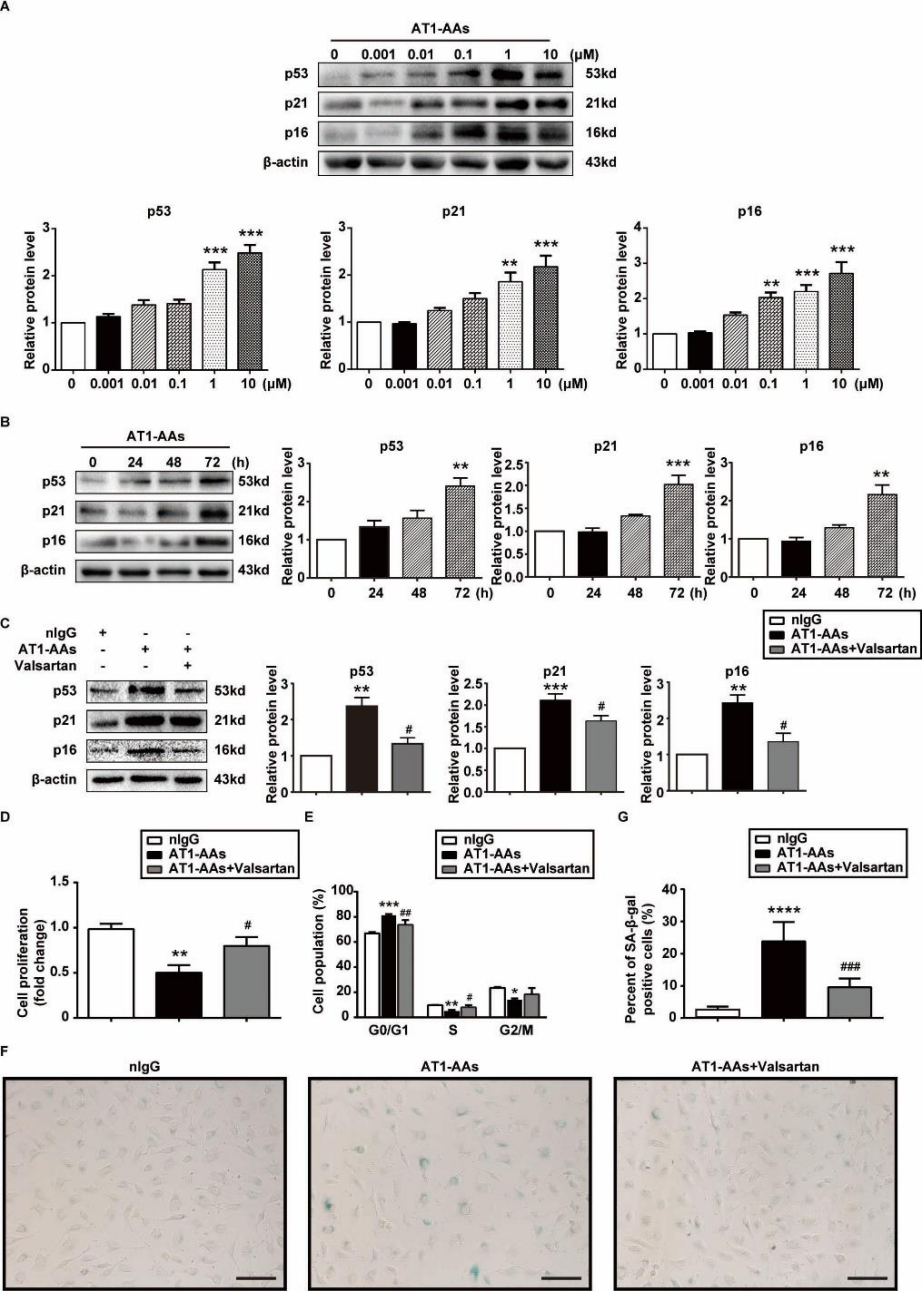
AT1-AAs induced premature senescence of HUVECs through AT1 receptor. A) Representative images and quantitative graphs of p53, p21 and p16^INK4a^ expressions. HUVECs were incubated with AT1-AAs-IgGs of indicated concentrations for 5 days. B) Representative images and quantitative graphs of p53, p21 and p16^INK4a^ expressions. HUVECs were incubated with AT1-AAs-IgGs (1 μM) for indicated times. **p < 0.01, ***p<0.001 *vs.* the control group. C) Representative western blot and quantitative graphs of p53, p21 and p16^INK4a^ expressions in HUVECs treated with nIgGs, AT1-AA or valsartan plus AT1-AA for 72 hrs. D. Measurement of cell proliferation using the cck-8 analysis. HUVECs treated with AT1-AA manifested a reduction in cell proliferation by comparison to the nIgG treated group. E) Cell cycle analysis of the nIgGs, AT1-AAs-IgGs or valsartan plus AT1-AAs-IgGs-induced HUVECs by flow cytometry. F) Photographs of typical SA-β-gal-stained HUVECs in the nIgGs, AT1-AA and valsartan+AT1-AA groups (senescent cells are stained blue). Scale bar = 400 μm. H. Quantification of percentages of SA-β-gal-positive HUVECs of the indicated groups. Data in the graphs were from 3 independent experiments and were expressed as mean ± SEM. *p < 0.05, **p < 0.01, ***p<0.001, ****p<0.0001 *vs.* the nIgGs group; #* p* < 0.05, ##* p* < 0.01, ###* p* < 0.001 *vs.* the AT1-AAs-IgGs group.

To clarify the pathological changes and characterize the progression of vascular aging, the aortas were harvested and subjected to hematoxylin-eosin staining. Significant thickening of the aortic wall was observed (Fig. 4A, B). Moreover, immunohistochemical staining for p53, p21 and p16^INK4a^ showed that AT1-AAs markedly enhanced expression of these aging-related molecules at the 12th week. Notably, the prominent increase was mainly distributed in the endothelial layer (Fig. 4C). Altogether, these results provided direct evidence that AT1-AAs caused vascular aging *in vivo*.

### AT1-AAs induced human umbilical vein endothelial cells (HUVECs) senescence via AT1 receptor

The total IgGs extracted from the immunized rats (termed AT1-AAs-IgGs) were loaded onto the sodium dodecyl sulfate polyacrylamide gel electrophoresis and then processed with Coomassie brilliant blue staining. The results showed that two strong bands were seen at 55 kDa and 25 kDa, representing the heavy and light chain of total IgGs, respectively (Supplementary Fig. 3A). To determine the activity of AT1-AAs-IgGs, the beating frequency of neonatal rat cardiomyocytes (NRCMs) was counted. As shown in Supplementary Fig. 3B, the AT1-AAs-IgGs strongly enhanced the beating frequency of NRCMs, which was inhibited by the AT1 receptor blocker, valsartan.

**Figure 6.**
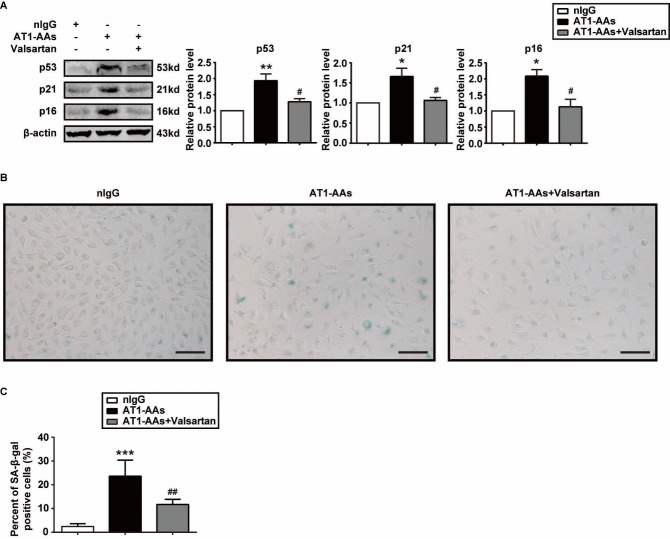
AT1-AAs from the PAD patients induced HUVECs senescence. A) Representative western blot and quantitative graphs of p53, p21 and p16^INK4a^ expressions in HUVECs treated with nIgGs, AT1-AAs-IgGs or valsartan plus AT1-AAs-IgGs for 72 hrs. B) Photographs of typical SA-β-gal-stained HUVECs in the nIgGs, AT1-AAs-IgGs and valsartan+AT1-AAs-IgGs groups. Scale bar = 400 μm. C) Quantification of percentages of SA-β-gal-positive HUVECs of the indicated groups. Data in the graphs were from 3 independent experiments and were expressed as mean ± SEM. *p < 0.05, **p < 0.01, ***p<0.001 *vs.* the nIgGs group; #* p* < 0.05, ##* p* < 0.01 *vs.* the AT1-AAs group.

**Table 3:** Logistic regression analysis.

Variables	B	Wald value	*P* value	OR	95%C.I. for OR	
Constant	-4.561	5.501	0.019	0.010		
AT1-AA (P/N)	1.269	10.284	0.001	3.559	1.638	7.730

B, partial regression coefficient; OR, odds ratio; C.l., confidence interval

To further investigate the association between AT1-AAs and vascular aging and vascular cells senescence, *in vitro* experiments were conducted with HUVECs. HUVECs were incubated with different concentrations (0, 0.001, 0.01, 0.1, 1 and 10 μM) of AT1-AAs-IgGs for 5 days. As shown in Fig. 5A, protein levels of p53, p21 and p16^INK4a^ were all spontaneously and persistently increased in cells treated with 1 μM of AT1-AAs-IgGs. To further explore the effect of AT1-AAs on these proteins, HUVECs were treated with AT1-AAs (1 μM) for various time intervals. The time-course experiment showed that AT1-AAs triggered a significant up-regulation of these senescent markers at 72 hrs (Fig. 5B). In contrast, no significant change was observed in the negative IgGs (nIgGs)-treated HUVECs (Supplementary Fig. 4A). In addition, in order to compare the inductive effect of Ang II on HUVECs senescence, cells were treated with Ang II (1 μM) and analyzed by western blot. A time-dependent upregulation was also observed. Unlike AT1-AAs, the initiative induction of Ang II was observed at 48 hrs (Supplementary Fig. 4B).

Cellular senescence is characterized by suppressed cell proliferation and permanent cell-cycle arrest. Our results showed that the proliferative activity was reduced in HUVECs treated with AT1-AAs-IgGs as compared with cells in the nIgGs-treated group (Fig. 5D). In addition, the rate of HUVECs arrested in the G0/G1 phase was higher and in the S/M phase was lower in the AT1-AAs-IgGs group (Fig. 5E). To examine the effect of AT1-AAs-IgGs on HUVECs senescence, senescence-associated β-galactosidase (SA β-gal) staining, a biomarker of cellular senescence, was performed. As shown in Fig. 5F, AT1-AAs-IgGs induced a high positive rate of SA β-gal, from 2.37±0.31 in nIgGs-treated HUVECs to 23.59±2.45 in cells treated with 1 μM of AT1-AAs-IgGs. Tumour necrosis factor alpha (TNF-α), interleukin (IL)-6 and monocyte chemoattractant protein (MCP)-1 are all important inflammatory factors involved in EC senescence. Herein, the mRNA levels of these factors were determined by RT-qPCR analysis. The results showed that AT1-AAs-IgGs increased the transcription of TNF α, IL-6 and MCP-1 drastically. In addition, treatment with AT1-AAs-IgGs enhanced the mRNA levels of intercellular adhesion molecule-1 (ICAM-1), vascular cell adhesion molecule-1 (VCAM-1) and plasminogen activator inhibitor (PAI)-1 (Supplementary Fig. 6). Finally, to determine whether the pro-senescent effect of AT1-AAs-IgGs was AT1 receptor-dependent,HUVECs were pre-incubated with valsartan (10 μM). As shown in Fig. 5C-G and Fig. 5, AT1-AAs-IgGs-induced upregulation of senescence markers, retardation of cell proliferation and cell cycle, SA β-gal-positive rate, as well as reduced phosphorylation of eNOS were almost completely blocked by valsartan. Besides, when cultured HUVECs were incubated with valsartan, the expression of the inflammatory molecules was significantly reduced (Supplementary Fig. 6). All these findings suggested that AT1-AAs-IgGs induced EC senescence, which was most probably through the AT1 receptor pathway.

### AT1-AAs from the PAD patients induced HUVECs senescence

AT1-AAs-IgGs were purified from the AT1-AAs-positive PAD patients, while nIgGs were from the non-PAD volunteers. The beating frequency of NRCMs was counted to determine the activity of AT1-AAs-IgGs. As Supplementary Fig. 7 shows, the AT1-AAs-IgGs markedly enhanced the beating frequency of NRCMs and could be inhibited by valsartan. Senescent markers expression and SA β-gal staining were performed to examine the effect of AT1-AAs-IgGs on HUVECs senescence. As shown in Fig. 6, AT1-AAs-IgGs induced increased expression of p53, p21 and p16^INK4a^ as well as a high positive rate of SA β-gal. The pro-senescent effect was mostly blocked by treatment with valsartan.

## DISCUSSION

The main findings of our research are (1) a high serum AT1-AAs level was closely associated with PAD; (2) long-term exposure to AT1-AAs could induce vascular aging and EC senescence *in vivo*; (3) AT1-AAs-IgGs induced EC senescence in an AT1 receptor-dependent pathway. We demonstrate for the first time that excess activation of AT1 receptor by its agonist autoantibody contribute to vascular aging and EC senescence, which provides important insights into the mechanism underlying vascular aging and EC senescence.

CVD are the leading cause of death worldwide. Aging of the large conduit arteries is a major cause of morbidity and mortality in the aging population. The aged arteries, characterized by complex structural and functional changes, are prone to hypertension, arterial calcification, atherosclerosis and increased stiffness. Autoantibodies against AT1 receptor, namely AT1-AAs, were first discovered in the serum of patients with pre-eclampsia [13]. Many studies have found that AT1-AAs exhibited an agonist-like activity like Ang II [24, 25]. In recent years, accumulating evidence suggested that AT1-AAs were involved in the pathogenesis of vascular injury [18, 26]. Besides, it has been shown that the AT1-AAs levels were correlated with aging phenotypes [20]. Based on the above, the question arises as to whether high serum levels of AT1-AAs play roles in accelerating vascular aging on the basis of increased cellular senescence. Our data showed that AT1-AAs provoked premature aging of the aortas and accelerated EC senescence. This finding provides a new clue about the association of elevated serum AT1-AAs level with CVD, and AT1-AAs may prove to be a hallmark hazard of endothelial senescence and atherogenesis.

A series of epidemiological studies have demonstrated that PAD is prevalent in the elderly. In clinical practice, PAD can be detected by the measurement of ankle-brachial index (ABI) and is diagnosed by the presence of an ABI < 0.9 [27]. The PAD is not just a disease of the peripheral arteries, but also is an indication of high probability of generalized vascular atherosclerosis [22] and other atherosclerotic diseases such as coronary artery disease and stroke [28]. This study examined the relationship between serum AT1-AAs level and PAD. The results revealed that in the PAD group, the positive rate of serum AT1-AAs was higher, and the levels were prevalently high, compared with the non-PAD volunteers. In comparison to non-PAD volunteers, PAD patients showed a high prevalence of common risk factors, including smoking history, hypertension, diabetes and dyslipidemia (Table 1, 2). Nevertheless, after adjustment for these factors, the serum AT1-AAs level was significantly associated with PAD (Table 3). These results suggested that serum AT1-AAs were closely related to vascular aging-related diseases.

Active immunization is a classic method for studying a variety of antibodies, which has been proven to be able to elicit autoantibodies and cause a similar syndrome as passive transfer of autoantibodies [19, 29]. Our previous research has demonstrated that AT1-AAs from the actively immunized rats and preeclamptic patients showed similar biological activity [18]. Our results showed that there were constant high levels of circulating antibodies throughout the study period in all rats of the immunized group, but not in the control rats (Fig. 2A). Therefore, antibodies in the current study could be considered as AT1-AAs.

To explore the role of AT1-AAs in vascular aging, we examined the time course of multiple noninvasive and invasive arterial physiological parameters and structural changes of the arteries in rats. Stiffening of large elastic arteries has emerged as a major independent risk factor for age-associated CVD [4]. In our study, AT1-AAs reduced carotid compliance with increased PWV. Besides, we demonstrated that endothelium-dependent vasodilatation of the immunized arteries was reduced. Actually, both of the above characteristics are thought to be key functional indicators of vascular aging and significantly contribute to CVD in the elderly [30]. eNOS, through generating the gasotransmitter nitric oxide (NO), plays an essential role in the regulation of endothelial function. Thus, eNOS acts as a master regulator of vascular tone and homeostasis. Dysregulation of the eNOS-NO axis induces endothelial dysfunction [31]. Herein, we found reduced eNOS phosphorylation of the aortas in the immunized rats (Supplementary Fig. 2). This suggests AT1-AAs might inhibit the activity of eNOS, but the mechanisms need to be further explored. During the process of immunization, we found that SBP began increasing at the 2 nd week and sustained to the 4 th week. However, the SBP showed no significant difference between the vehicle and immunized groups from the 6th week. Consistent with our findings, SBP was unchanged in the study by Wang et al [26]. It might be because that blood pressure in the immunized rats was adjusted to normal by other antihypertensive mechanisms, or due to decreased cardiac function by AT1-AAs as previous study confirmed [32].

Endothelial damage has been recognized as the key step in the pathophysiology of diverse cardiovascular abnormalities associated with aging. Vascular cell senescence, especially EC, is involved in vascular aging [33]. EC senescence has been recognized to be linked to aging-related endothelial dysfunction and atherosclerosis [34]. Thus, the slowing of EC senescence is emerging as an exciting possibility for controlling vascular diseases. In the present study, we demonstrated for the first time that AT1-AAs could induce vascular aging, which is considered the early event in the development of atherosclerosis. Especially, AT1-AAs exposure caused endothelial dysfunction and endothelial senescence *in vivo*. Besides, our *in vitro* data suggested that AT1-AAs-IgGs extracted from the serum of immunized rats induced EC senescence, even in the absence of other environmental injury stimuli, and could be blocked by the AT1 receptor blocker. Our findings that AT1-AAs treatment caused HUVECs senescence *in vitro* is consistent with spontaneous vascular aging in AT_1_R-EC_II_-immunized rats. Cellular senescence was initially described as the limited ability of somatic cells to divide when cultured *in vitro*, which was termed as replicative senescence [35]. More recently, it has been increasingly realized that EC senescence can also be induced by a number of stress stimuli and thus leads to stress-induced premature senescence (SIPS) [36]. Compared with the replicative senescence, usually accompanied by telomere shortening, SIPS seems to be particularly relevant to vascular aging, in which the vessel wall-resident ECs may not be able to replicate sufficiently [37].

Numerous studies have demonstrated that activation of the AT1 receptor plays vital roles in regulating the aging processes of the vascular system [38]. Recently discovered autoantibodies against AT1 receptor (AT1-AAs) might provide some new ideas about the complicated pathological process. The main mechanism of the agonistic effect involves the molecular mimicry theory, where AT1-AAs specifically bind to the second loop of the AT1 receptor and activate it [13]. Accumulating evidence indicates that AT1-AAs induce several senescence-associated signaling mechanisms, including reactive oxygen species (ROS) production, inflammatory response and NF-κB activation by activating the reduced form of nicotinamide adenine dinucleotide phosphate (NADPH) oxidase (Nox) [16, 39]. Oxidative stress has been considered a key driver of the aging process, and subsequent studies have revealed the link between oxidative stress and senescence [40]. It has been shown that ROS contributes to endothelial senescence by decreasing NO production, promoting inflammation and perturbing ECs functions [41, 42]. Nox, which produces ROS, is pivotal to EC senescence and the pathophysiology of various vascular diseases [43, 44]. Inflammation is recognized as a primary pathological mechanism of aging-related endothelial dysfunction and aging of arteries [45]. The serum concentrations of inflammatory proteins increase with age, including pro-inflammatory cytokines such as TNF-α, IL-6 and MCP-1. Therefore, the upregulation of these cytokines with AT1-AAs treatment reflects a chronic pro-inflammatory state correlated with aging. Such a condition is attributable to NF-κB activation [46]. Indeed, NF-κB has been reported to be activated and promote gene transcription of pro-inflammatory cytokines after AT1-AAs treatment [17]. Moreover, NF-κB gene expression may result in increased levels of ICAM-1 and VCAM-1. AT1-AAs may also contribute to the surface adhesion molecule expression and tissue factor production [47, 48]. In addition to the above possibilities, AT1-AAs might induce EC senescence *via* the activation of small G-proetin Ras, mitogen-activated protein kinases and other transcription factors such as activator protein (AP)-1, all of which are the signaling cascades downstream of AT1 receptor activation [49].

Under the conditions of the same total doses, the AT1-AAs-induced EC senescence occurred later than that of Ang II (Supplementary Fig. 4B), indicating a relative weaker inductive effect of AT1-AAs. Nevertheless, the specific reasons for this need to be further explored. Unlike the natural ligands, the agonistic autoantibodies are able to promote a sustained receptor activation without the customary desensitization [50, 51]. Thus, the detrimental effects of AT1-AAs may be attributed to the excessive AT1 receptor activation. Beyond that, a previous study has shown that AT1-AAs might increase the sensitivity of Ang II in ECs [52].

In summary, our findings show a novel pathological factor contributing to vascular aging, especially EC senescence. The increasing level of serum AT1-AAs should be considered as a clinical assessment factor in the deterioration of vascular-aging related diseases.

## Supplementary Materials

The Supplemenantry data can be found online at: www.aginganddisease.org/EN/10.14336/AD.2018.0919.
